# Timing of carbohydrate ingestion did not affect inflammatory response and exercise performance during prolonged intermittent running

**DOI:** 10.1186/s40064-016-2108-6

**Published:** 2016-04-23

**Authors:** Sahiro Mizuno, Chihiro Kojima, Kazushige Goto

**Affiliations:** Graduate School of Sports and Health Science, Ritsumeikan University, 1-1-1, Nojihigashi, Kusatsu, Shiga 525-8577 Japan; Faculty of Sports and Health Science, Ritsumeikan University, 1-1-1, Nojihigashi, Kusatsu, Shiga 525-8577 Japan

**Keywords:** Carbohydrate, Inflammatory response, Prolonged exercise, Interleukin-6

## Abstract

**Background:**

Carbohydrate ingestion during exercise is known to attenuate exercise-induced elevation of plasma IL-6 concentration. However, the influence of timing of carbohydrate ingestion remains unclear.

**Purpose:**

The present study investigated the influence of different timing of carbohydrate ingestion during a simulated soccer game on exercise performance, metabolic and inflammatory responses.

**Methods:**

Seven active males performed 3 exercise trials in a randomized order. The exercise consisted of two consecutive bouts of 45 min running (4–16 km/h), separated with 15 min rest period between bouts. The subjects ingested carbohydrate gel (1.0 g/kg) immediately before the first bout of exercise (ONE), immediately before first and second bouts of exercise (0.5 g/kg for each ingestion) (TWO) or placebo immediately before exercise (PLA) Time course changes of maximal jump height, peak power output during 6-s maximal pedaling, perceived fatigue and heart rate (HR) were monitored. Blood samples were also drawn to determine blood glucose, serum insulin, free fatty acid (FFA), myoglobin (Mb), creatine kinase (CK) and plasma IL-6 concentrations.

**Results:**

Blood glucose and serum insulin concentrations were significantly higher in the ONE trial after first bout of 45 min exercise compared with PLA trial (*P* < 0.05), while serum FFA concentration was significantly elevated in PLA compared with ONE and TWO trials after second bout of exercise (*P* < 0.05). However, changes of jump height, peak power output during 6-s maximal pedaling, perceived fatigue, HR, or indirect muscle damage (Mb, CK) and inflammatory (IL-6) markers were not significantly different among three trials (*P* > 0.05).

**Conclusions:**

The timing of carbohydrate ingestion did not affect exercise performance, exercise-induced muscle damage or inflammatory response during a simulated soccer game.

## Background

Both strenuous strength exercise (e.g., resistance exercise, rebound jump) and prolonged endurance exercise (e.g., marathon running) involving eccentric contraction result in exercise-induced muscle damage (EIMD) (Nosaka et al. 1991; Child et al. 1998; Nosaka and Sakamoto 2000). The majority of previous studies evaluated time courses changes in various symptoms of EIMD, including muscular strength loss (Clarkson and Sayers 1999), reduction of range of motion (Chen et al. 2009), swelling of muscle (Hirose et al. 2004), decrease in running economy (Satkunskienė et al. 2015) and intracellular enzyme or proteins in blood circulation (Peake et al. 2005). Among them, elevated concentrations of creatine kinase (CK) and myoglobin (Mb), and inflammatory cytokines are known to be prevalent parameters as indirect markers of EIMD (Nehlsen-Cannarella et al. 1997; Nieman et al. 2003; Kłapcińska et al. 2013; Del Coso et al. 2013). Prolonged running causes marked elevation of plasma interleukin-6 (IL-6) concentration (Nieman et al. 2014). The elevated IL-6 concentration mainly reflects locally produced cytokine from working muscles (Ostrowski et al. 1998). Del Coso et al. (2013) reported that declining of running speed during a full marathon was positively correlated with elevation of serum Mb concentration. In addition, the games of team sports such as soccer and basketball which involve highly metabolic demands evoke EIMD (Souglis et al. 2015; Chatzinikolaou et al. 2014). In particular, soccer matches have been shown to produce profound muscle damage compared with the other type of team sports (Souglis et al. 2015). During soccer matches, the players are required to cover large distances under high velocity, and average heart rate (HR) during the game was reported to be equivalent to about 86.9 ± 4.3 % for maximal HR (Souglis et al. 2015). In addition, various activities such as jump, agility and change of direction are frequently observed over 90 min of game. Furthermore, CK concentration was increased twice, and IL-6 concentration was elevated by four–five-fold following soccer game (Souglis et al. 2015). Inflammatory cytokine release such as IL-6 during intensive exercise is suggested to be a factor for reduction of exercise performance (Welc and Clanton 2013). Collectively, attenuation of muscle damage and inflammatory response during team sports is considered to be important to delay the onset of fatigue and to maintain exercise capacity throughout the game.

There are abundant evidences to support that carbohydrate ingestion during prolonged exercise reduces inflammatory (e.g., IL-6) response (Bishop et al. 2002; Nieman et al. 2003). Plausible reason for reduced IL-6 concentration by exogenous carbohydrate supply during exercise linked to muscle glycogen content (Helge et al. 2003). In a situation with depletion of muscle glycogen content (e.g., during prolonged running), IL-6 was markedly produced to stimulate liver glycogenolysis and gluconeogenesis (Blumberg et al. 1995). Therefore, providing carbohydrate during exercise maintains blood glucose concentration and spares muscle glycogen content, leading to attenuation of IL-6 production. Although the effect of carbohydrate ingestion on maintenance of blood glucose concentration during prolonged exercise is more apparent (Nehlsen-Cannarella et al. 1997; Bishop et al. 2002; Kohara et al. 2014), influence of different timing of carbohydrate ingestion during team sports game has not been clarified. Sugiura and Kobayashi (1998) demonstrated that ingestion of glucose polymer solution at middle of the 90-min of high-intensity pedaling exercise improved pedaling power output during second half of the exercise compared with placebo ingestion. However, the above study by Sugiura has not focused on influence of different timing of carbohydrate ingestion on EIMD and inflammatory response.

Therefore, the present study determined the influence of different timing of carbohydrate ingestion during prolonged running (a simulated soccer game) on EIMD and inflammatory responses. To accomplish this purpose, we compared the effect of identical amount of carbohydrate gel ingestion with different timing (immediately before exercise vs. immediately before exercise and halftime). We hypothesized that carbohydrate ingestion would maintain blood glucose concentration and attenuate EIMD and inflammatory response despite different timing of ingestion.

## Methods

### Subjects

Seven men [mean ± standard error (SE): age, 21.9 ± 0.6 years; height, 171.3 ± 1.1 cm; body mass, 59.1 ± 1.1 kg] were recruited in the present study. They were physically active, with regular exercise habit at least 2 days a week. Exclusion criteria included a history of inflammatory conditions and musculoskeletal injuries. Smokers and individuals with taking antioxidant supplements were also excluded. All of the subjects gave informed consent after being informed of the purpose and risks associated with the study. This study was approved by the Ethics Committee of the Ritsumeikan University, Japan.

### Experimental design

The present study was conducted with a placebo-controlled, double-blinded design. Subjects performed 3 exercise trials with three nutritional manipulations. Experimental trials were conducted in a random order, which were separated by approximately 7 days. The exercise trial consisted of (1) carbohydrate gel ingestion (1.0 g/kg) immediately before the first bout of exercise (ONE); (2) carbohydrate gel ingestion immediately before first and second bouts of exercise (0.5 g/kg for each ingestion) (TWO); and (3) placebo ingestion (timing and the amount of volume consumed were equivalent to the ONE trial) immediately before exercise (PLA). The carbohydrate gel comprised dextrin. Each gel was packed by identical aluminum bag, and the subjects reported that the gels were indistinguishable by taste and texture. The placebo gel was prepared by company (Morinaga & CO., LTD., Tokyo, Japan) with same texture and flavor using artificial sweetener without dextrin (total caloric value was 0 kcal).

### Exercise protocol

**Fig. 1:**
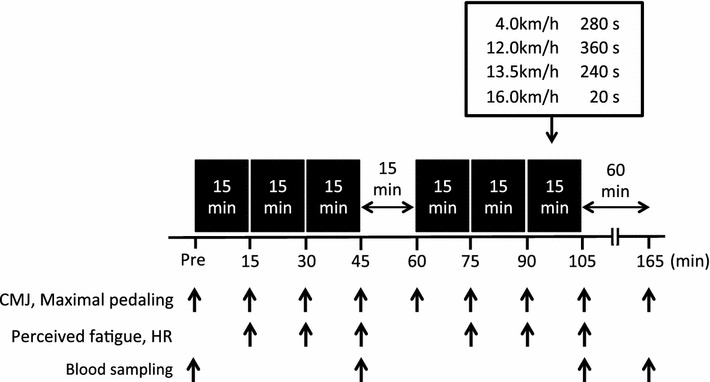
Schematic illustration of the time course of measurements for counter movement jump (CMJ), maximal pedaling, perceived fatigue, heart rate (HR) and blood variables

On each experimental trial day, the subjects arrived at the laboratory after an overnight fast and rested for 30 min before the first blood collection. The blood samples were drawn from an antecubital vein, and subjects performed maximal jump height and 6-s maximal pedaling measurements to determine baseline values. After completing these baseline measurements, subjects consumed the carbohydrate or placebo gel immediately before the exercise. The exercise protocol consisted of consecutive two bouts of 45 min running (90 min in total) on treadmill (Elevation series E95Ta; Life Fitness Corporation, Japan), separated with rest period of 15 min between bouts (Fig. 1). Each bout further consisted of 3 × 15 min running. The running speed during each 15 min session was progressively increased ranging from 4.0 to 16.0 km/h. The exercise protocol in the present study was designed based on previously reported protocol by Abbey and Rankin (2009) with some modifications, to simulate a soccer match. The subjects were allowed to drink 750 ml water throughout the exercise. The room temperature was maintained at 23.0 °C.

Maximal jump height and peak power output during 6-s maximal pedaling were evaluated before exercise, during exercise (every 15 min) and 60 min after exercise. These measurements were completed within 2 min, and duration of measurement was not included in 90 min of exercise duration. Perceived fatigue (VAS) and heart rate (HR) was also assessed during exercise (every 15 min). Blood samples were collected before exercise, immediately after each bout of 45 min exercise and 60 min after exercise.

### Jump performance

The subjects performed a maximal jump on a platform (Multi jump tester; DKH corporation, Japan) that was connected to a computer. They were instructed to conduct as high as possible while placing hands on the lumbar division to eliminate upper-limb effect. The flight time during vertical jump was recorded. From the flight time, the CMJ height was calculated using the following formula. Jump height (cm) = 1/8 (flight time)^2^ × the gravity constant (=9.81 m/s^2^).

### Maximal pedaling

Peak power output during 6-s maximal pedaling was measured using a cycle ergometer (Power max VIII; Combi Wellness Corporation, Tokyo, Japan). The resistance was set at 6.0 % of each subject’s body mass. The peak power output during 6-s maximal pedaling was recorded and used for further analysis.

### Perceived fatigue

Perceived fatigue was assessed using a 10 cm visual analog scale, with 0 cm indicating no fatigue and 10 cm indicating the worst fatigue experienced. The subjects were familiarized with the scale before the measurement.

### Blood analysis

Initial blood sample was taken from the antecubital vein after 30 min of rest. Blood samples were used to measure blood glucose, lactate, serum insulin, and free fatty acid (FFA), creatine kinase (CK), myoglobin (Mb) and plasma IL-6 concentrations. Serum and plasma samples were obtained by centrifuging at 3000 rpm for 10 min at 4 °C. The plasma and serum samples were stored at −60 °C until analysis. Blood glucose and lactate concentrations were measured using an automatic glucose analyzer (Free style, Nipro Corporation, Osaka, Japan) and lactate analyzer (Lactate pro, Arkray Inc, Kyoto, Japan), respectively. Serum insulin and FFA concentrations were measured using chemiluminescent enzyme immune assays (Fujirebio Inc., Tokyo, Japan) at a clinical laboratory (SRL Inc., Japan). Serum CK and Mb concentrations were also measured at the SRL Clinical Laboratory in Tokyo, Japan. The plasma IL-6 concentration was assayed with an enzyme-linked immunosorbent assay (ELISA) kit (R&D Systems, Minneapolis, MN, USA). The intra-assay coefficients of variation for each measurement were as follows: 3.1 % for insulin, 1.3 % for FFA, 2.8 % for CK, 2.4 % for Mb and 6.6 % for IL-6.

### Statistical analysis

Data are expressed as mean ± SE. Time course of changes in exercise performance, blood parameters and muscle soreness were initially analyzed using two-way analysis of variance (ANOVA) with repeated measures. When the ANOVA revealed a significant interaction or main effect, the Tukey–Kramer post hoc test was applied to identify the differences. The significance level was set at *P* < 0.05.

## Results

Time course changes in blood glucose, lactate, and serum FFA concentrations are presented in Table 1. Blood glucose concentration was significantly higher in ONE trial compared with PLA trial after first bout of 45 min exercise (trial × time interaction: *P* < 0.05, η^2^ = 0.44). However, no significant difference was observed between TWO and PLA trials at any time point. Blood lactate concentration increased significantly during exercise in all three trials (main effect for time: *P* < 0.05, η^2^ = 0.58). However, there was no significant interaction (trial × time) (η^2^ = 0.15) or main effect for trial (η^2^ = 0.15). Serum FFA concentration was significantly higher in PLA compared with in ONE and TWO trials after second bout of 45 min (main effect for trial: *P* < 0.05, η^2^ = 0.57). Furthermore, PLA trial showed significantly higher value than TWO trial at 60 min after exercise (*P* < 0.05). In the ONE and TWO trials, serum FFA concentration did not change significantly over time (*P* > 0.05 vs. Pre).

**Table 1:** Blood glucose, lactate and serum FFA concentrations in each trial

	Pre	After first bout of 45 min	After second bout of 45 min	60 min after exercise
Glucose (mg/dl)				
ONE	94 ± 3	106 ± 7^†^	82 ± 9	80 ± 1*
TWO	91 ± 2	96 ± 3	91 ± 3	76 ± 4
PLA	90 ± 2	86 ± 3	85 ± 4	80 ± 1*
Lactate (mmol/1)				
ONE	1.3 ± 0.9	5.3 ± 1.8*	4.6 ± 1.5	1.8 ± 0.2
TWO	1.3 ± 0.1	5.0 ± 1.5*	4.2 ± 1.3*	1.8 ± 0.1
PLA	1.3 ± 0.1	4.8 ± 1.4*	4.7 ± 1.4*	2.2 ± 0.5
FFA (nEq/1)				
ONE	330 ± 46	192 ± 31	334 ± 57^†^	525 ± 135
TWO	314 ± 57	232 ± 46	455 ± 88^†^	441 ± 75
PLA	367 ± 57	309 ± 44	678 ± 50*	826 ± 155*^‡^

Values are mean ± SE

* *P* < 0.05 versus Pre; ^†^ *P* < 0.05 versus PLA; ^‡^ *P* < 0.05 versus TWO

**Fig. 2:**
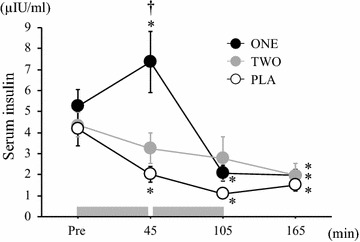
Changes in serum insulin during exercise period in each trial. Values are mean ± SE. The *gray bar* indicates the duration of exercise. ^†^ *P* < 0.05 versus TWO and PLA. *P < 0.05 versus Pre

Figure 2 represents time course of changes in serum insulin concentration. There was a significant interaction (trial × time) (η^2^ = 0.47) and main effects for trial (η^2^ = 0.60) and time (*P* < 0.05, η^2^ = 0.72). After first bout of 45 min exercise, serum insulin concentration was significantly higher in ONE trial compared with other two trials (*P* < 0.05), whereas no significant difference was observed between TWO or PLA trials at any time point.

**Fig. 3:**
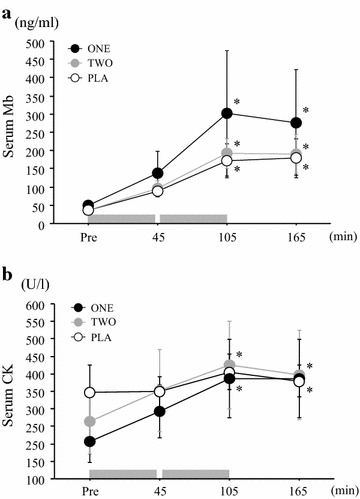
Changes in serum Mb (**a**) and CK (**b**) throughout exercise period in each trial. Values are mean ± SE. The *gray bar* indicates the duration of exercise *P < 0.05 versus Pre

Figure 3a, b represents time course of changes in serum Mb and CK concentrations in each trial. All trials showed significant increases in serum Mb and CK concentrations during exercise (main effect for time: *P* < 0.05, Mb: η^2^ = 0.75, CK: η^2^ = 0.41). However, there was no significant difference among the three trials at any time point for Mb or CK (trial × time interaction: *P* > 0.05 Mb: η^2^ = 0.04, CK: η^2^ = 0.14, main effect for trial: *P* > 0.05 Mb: η^2^ = 0.01, CK: η^2^ = 0.10).

**Fig. 4:**
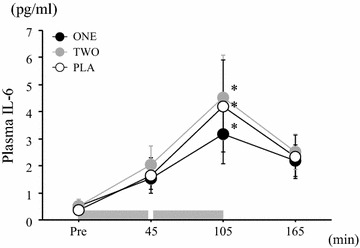
Changes in plasma IL-6 throughout exercise period in each trial. Values are mean ± SE. The *gray bar* indicates the duration of exercise *P < 0.05 versus Pre

As shown in Fig. 4, plasma IL-6 concentration was significantly elevated with the exercise in all trials (main effect for time: *P* < 0.05, η^2^ = 0.54). However, the response was not significantly different among trials (trial × time: *P* > 0.05, η^2^ = 0.23, main effect for trial: *P* > 0.05, η^2^ = 0.15).

**Fig. 5:**
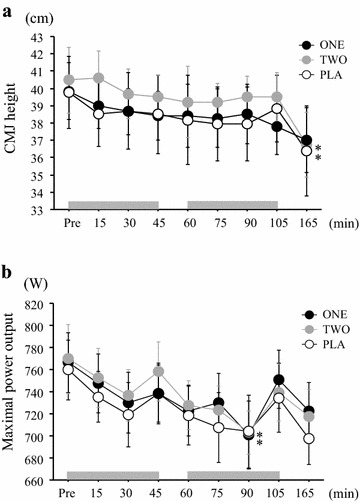
Changes in CMJ height (**a**) and power output during 6 s of maximal pedaling (**b**) throughout exercise period in each trial. Values are mean ± SE. The *gray bar* indicates the duration of exercise *P < 0.05 versus Pre

During exercise, time course changes in CMJ height and peak power output during 6-s maximal pedaling were evaluated (Fig. 5a, b). The CMJ height significantly decreased during exercise in all trials (main effect for time: *P* < 0.05, η^2^ = 0.39). However, there was no significant interaction (trial × time) (η^2^ = 0.11) or main effect for trial (η^2^ = 0.15). Peak power output during 6-s maximal pedaling also decreased significantly during exercise (*P* < 0.05, η^2^ = 0.30), but no significant difference was observed among all trials at any time point (trial × time: *P* > 0.05, η^2^ = 0.09).

Table 2 presents time course changes in scores of fatigue and HR during exercise. Although the scores of perceived fatigue increased gradually with progress of exercise in each trial (main effect for time: *P* < 0.05, η^2^ = 0.76), there was no significant interaction (trial × time) (η^2^ = 0.10) or main effect for trial (η^2^ = 0.003). In addition, HR during exercise did not show significant difference among all trials at any time point (trial × time: *P* > 0.05, η^2^ = 0.17, main effect for trial: *P* > 0.05, η^2^ = 0.29).

**Table 2:** Score of perceived fatigue and heart rate during exercise

	Pre	First bout of 45 min			Second bout of 45 min		
		15 min	30 min	45 min	15 min	30 min	45 min
Perceived fatigue							
ONE	2.7 ± 6.0	4.7 ± 0.6*	4.5 ± 0.8*	5.7 ± 0.7*	5.7 ± 0.8*	6.5 ± 0.7*	6.7 ± 0.7*
TWO	2.6 ± 6.4	4.2 ± 0.6	5.0 ± 0.7*	5.5 ± 0.7*	5.8 ± 0.6*	6.6 ± 0.5*	7.0 ± 0.7*
PLA	2.5 ± 5.9	5.1 ± 0.4*	4.6 ± 0.7*	5.5 ± 0.7*	5.5 ± 0.6*	6.8 ± 0.5*	6.8 ± 0.5*
HR (bpm)							
ONE	–	162 ± 7	166 ± 6	168 ± 6	166 ± 6	167 ± 7	170 ± 6
TWO	–	167 ± 7	168 ± 6	169 ± 6	169 ± 6	172 ± 6	173 ± 6
PLA	–	165 ± 7	167 ± 6	169 ± 6	167 ± 6	169 ± 6	170 ± 6

Values are mean ± SE

* P < 0.05 versus Pre

## Discussion

The present study investigated the effect of different timing of carbohydrate ingestion during prolonged intermittent running on EIMD and inflammatory responses. As we expected, blood glucose concentration was elevated with carbohydrate ingestion (1.0 g/kg) before 90 min of a simulated soccer game. However, the supply of carbohydrate gel during exercise session did not attenuate exercise-induced elevations of CK, Mb or IL-6 concentrations with independent of frequency of ingestion. Similarly, no significant difference was observed in time course changes in exercise performance, HR or perceived fatigue among trials.

In the present study, blood glucose and serum insulin concentrations were significantly elevated in the ONE trial after first 45 min bout of exercise. However, these concentrations recovered completely to baseline levels immediately after second bout of 45 min exercise. As muscle contraction itself stimulates glucose uptake via independent pathways from insulin action (Wasserman et al. 1991), onset of the exercise accelerates blood glucose uptake into the working muscle. In contrast, plasma FFA concentration in PLA trial was significantly elevated compared with other 2 trials with carbohydrate ingestion. These results indicated that fat metabolism was attenuated in the ONE and TWO trials, which was associated with supplying glucose during exercise.

Previous studies reported that continuous ingestion of carbohydrate before or during prolonged exercise attenuated elevation of plasma IL-6 concentration (Nehlsen-Cannarella et al. 1997; Nieman et al. 2005). However, beneficial effect of carbohydrate ingestion was not evident in the present study. The finding is consistent with previous studies by Abbey and Rankin (2009) and Sim et al. (2012). Abbey and Rankin (2009) reported that carbohydrate ingestion (1.0 g/kg) before and the middle of 90 min of a simulated soccer game did not influence plasma IL-6 concentration. Similarly, carbohydrate ingestion (3 ml/kg; 6 % carbohydrate solution) during 90 min of running did not affect exercise-induced IL-6 response (Sim et al. 2012). The author speculated that the lack of the influence of carbohydrate ingestion on inflammatory response was due to insufficient amount of carbohydrate (a total of 855 ml solution, containing 0.73 g/kg of carbohydrate). However, the volume consumed during exercise session (1.0 g/kg) in the present study does not appear to be problematic because similar volume of carbohydrate (a total of 1219 ml solution, containing 1.1 g/kg of carbohydrate) revealed previously attenuation of inflammatory response during 90 min of high-intensity intermittent running (Bishop et al. 2002). Therefore, inconsistent outcomes among relevant studies may be attributed to the timing of carbohydrate consumption. In the previous studies that reported beneficial effect of carbohydrate ingestion (Nehlsen-Cannarella et al. 1997; Nieman et al. 2005), the subjects were provided carbohydrate frequently during prolonged exercise (every 15 min). Similarly, Bishop et al. (2002) demonstrated that carbohydrate ingestion during intermittent shuttle running maintained blood glucose concentration and subsequently blunted elevation of IL-6 concentration when the carbohydrate was supplied every 15 min during the exercise. Considering these results, it is possible that more frequent supplementation of carbohydrate may cause apparent protective effect on elevation of IL-6 concentration. Furthermore, Mb and CK concentrations (indirect muscle damage markers) did not differ significantly among three trials in the present study, although we expected that carbohydrate ingestion before and during exercise attenuated elevation of muscle damage markers. A plausible reason for lack of the effect may be also lower frequency of carbohydrate ingestion during exercise sessions.

Inflammatory cytokine (e.g., IL-6) release during intensive exercise is suggested to be a factor for reduction of exercise performance (Welc and Clanton 2013). In the present study, we monitored time courses of changes in CMJ and power output during 6-s maximal pedaling to evaluate exercise capacity. Previous studies reported that carbohydrate ingestion significantly improved exercise performance during a simulated soccer game (Russell et al. 2012; Phillips et al. 2010; Ali and Williams 2009). Phillips et al. (2010) reported that running capacity was significantly improved when carbohydrate solution (5 ml/kg before exercise, 2 ml/kg during exercise; 6 % maltodextrin solution) was ingested before exercise and every 15 min during the exercise. However, they did not observe any beneficial effect of carbohydrate ingestion on sprint running performance. Similarly, we were not able to detect efficacy of carbohydrate ingestion for anaerobic power output (e.g., CMJ height and maximal pedaling power output). These results suggest that carbohydrate ingestion might have relatively small impact on maximal anaerobic performance (explosive power output) during prolonged exercise. During maximal anaerobic performance within several seconds, the energy required is mainly provided via ATP–PCr system. In addition, carbohydrate ingestion might improve central nervous function and attenuate the impairment of motor skill performance (Bandelow et al. 2010). However, we did not evaluate skill performance during the exercise. Therefore, future studies are needed to monitor effect of carbohydrate ingestion on motor skills and central fatigue in addition to changes in anaerobic and aerobic exercise performance.

Several limitations should be carefully considered in the present study. First, we did not prepare the control trial in which the subjects consumed placebo with the same timing as TWO trial. Therefore, ingestion of carbohydrate or placebo was not blinded completely for TWO trial. Second, although we have monitored time courses of changes in exercise performance during several seconds, we were not able to determine anaerobic endurance performance mainly carried out via glycolytic energy system to avoid severe fatigue. Finally, we recruited physically active males to increase repeatability for performance variables. However, inter-individual variations were found for several variables due to relatively small sample size. From a practical point of view, carbohydrate ingestion with relatively low frequency might not be an appropriate strategy to attenuate EIMD and inflammatory response during games of team sports, and frequent carbohydrate ingestion during the game may be recommended.

## Conclusions

A single carbohydrate ingestion (1.0 g/kg) immediately before 90 min of prolonged intermittent running resulted in higher blood glucose concentration, whereas separated ingestion before exercise and the middle of 90 min exercise did not increase blood glucose or serum insulin concentration. Furthermore, the timing of carbohydrate ingestion did not affect exercise performance, EIMD or inflammatory response.

## Abbreviations

ANOVA: analysis of variance
CK: creatine kinase
CMJ: counter movement jump
EIMD: exercise-induced muscle damage
FFA: free fatty acid
IL-6: interleukin-6
HR: heart rate
Mb: myoglobin
VAS: visual analog scale
